# New Global Referencing Approach in a Camera-LCD Micro Positioning System

**DOI:** 10.3390/s20072118

**Published:** 2020-04-09

**Authors:** Óscar de Francisco Ortiz, Irene Ortiz, Antonio Bueno

**Affiliations:** 1 Department of Engineering and Applied Technologies, University Center of Defense, San Javier Air Force Base, MDE-UPCT, 30720 Santiago de la Ribera, Spain; 2 Department of Science and Computer Science, University Center of Defense, San Javier Air Force Base, MDE-UPCT, 30720 Santiago de la Ribera, Spain; irene.ortiz@cud.upct.es; 3 Department of Geometry and Topology, University of Granada, 18071 Granada, Spain; jabueno@ugr.es

**Keywords:** accuracy, pattern recognition, positioning, artificial vision, precision

## Abstract

In any precision manufacturing process, positioning systems play a very important role in achieving a quality product. As a new approach to current systems, camera-LCD positioning systems are a new technology that can provide substantial improvements enabling better accuracy and repeatability. However, in order to provide stability to the system a global positioning system is required. This paper presents an improvement of a positioning system based on the treatment of images on an LCD in which a new algorithm with absolute reference has been implemented. The method is based on basic geometry and linear algebra applied to computer vision. The algorithm determines the spiral center using an image taken at any point. Consequently, the system constantly knows its position and does not lose its reference. Several modifications of the algorithm are proposed and compared. The simulation and test of the algorithm provide an important improvement in the reliability and stability of the positioning system providing errors of microns for the calculation of the global position used by the algorithm.

## 1. Introduction

Recent studies have demonstrated the benefits of using computer vision systems for automatic machine tool control. In fact, the toolpath optimization in a Computer Numerical Control machine (CNC machine) was studied by Ahmad et al. [1] using image processing algorithms. More recently, Zhang et al. [2] estimated the position of the cutting tool by reconstructing a 3D image using a single camera. In addition, to protect the machines against collisions between tools and fasteners, Karabagli et al. [3] developed a vision system for automatic verification of machining accessories. In the context of micromachining, Chen et al. [4] proposed a machine method to measure and compensate for errors caused by the inaccuracy of mechanical systems. Furthermore, different closed loop positioning systems that analyze the patterns shown in the image of the LCD screen have been developed, achieving a certain degree of accuracy using different approaches such as in Wong [5], Montes [6], Leviton [7] and more recently by de Francisco [8,9].

Identifying the same points in two images to control the position is essential in the analysis of the movement controlled by vision. The calculation of the movement of an object from a sequence of two-dimensional images has been extensively analyzed and discussed by Roach [10] and Yachida [11] using two frames of a sequence to solve this problem. Later Sethi [12] used a sequence of frames to explore the smoothness of the motion by proposing an iterative algorithm to find trajectories of points in a monocular image sequence. Ramírez-Hernández [13] proposes a novel camera calibration method to improve the accuracy of stereo vision systems for three-dimensional point localization. In his approach, the least square method was used to model the error caused by the image digitization and the lens distortion with two images taken by two different cameras. Other machine vision systems have been used to experimental measurement of velocity as in Valenzuela-Delgado [14], Castro-Toscano [15,16].

The use of geometric and symmetric patterns in a vision system provides a good local positioning system, which requires additional external information to achieve absolute positioning successfully [9] or depends on the previous relative positions assuming the cumulative errors generated. This manuscript presents a step forward creating an asymmetric and non-periodic pattern so that each image taken is unique. A spiral pattern, including the conditions mentioned, is presented as a solution. Pattern analysis using the artificial vision algorithm will be more difficult than the analysis required by using a symmetric pattern but will provide better results in the overall positioning system.

The algorithm used for the analysis was initially created using Matlab [8]. Theodoridis [17] describes how to perform pattern recognition using Matlab. The solution presented in this work consists of the use of a logarithmic spiral as a geometric base to calculate the global positioning. Mathematical aspects of the logarithmic spiral have been studied by Catrakis [18], who clarifies and summarizes years of mathematical studies on spirals considering mathematical aspects of the logarithmic spiral and its utility in turbulence modeling. Logarithmic spirals have also been analyzed and used in image processing, but with different approaches as summarized below.

Weiman [19] presented a picture digitization grid based on logarithmic spiral coordinates but focused on edge detection applied to large images. Later, Rojer [20] discussed about a space-variant sensor design based on the conformal mapping of the half disk, which characterizes the anatomical structure of the primate, and human visual systems. He presented an analysis which makes it possible to compare directly the space complexity of different sensor designs in the complex logarithmic family. More recently, Palacios [21] proposed an image processing for computer vision based on a combined map. This method was applied to a model on the workings of visual cortical area which attributes are approximately logarithmic at the periphery. Finally, Zhao [22] discussed some of the main features of biological eagle-eye vision technology, providing a study about the eagle-eye and its relation with visual information processing technology, including the logarithmic spiral movement of the eagle and the eagle-eye visual attention mechanism.

The work presented below is based on the calculation of a logarithmic spiral center with three different methods using the same mathematical background, based on the treatment of images on an LCD in which a new algorithm with absolute reference has been implemented. As will be shown, a comparison has been made between the algorithms developed for each method to determine which provides better consistency and accuracy during position determination. The most accurate method presents an important improvement in the reliability and stability of the positioning system. As novelty, the simulation and test provide errors of microns for the calculation of the global position used by the algorithm.

## 2. Methodology

The need for a global reference coordinate system to accurately determine the position is, as already introduced, a necessary requirement to achieve stability and reliability in the positioning system. In addition, one of the main problems of the system presented and developed in the previous related works, due to the use of a repetitive pattern on the LCD screen, is the inability of the system to know exactly its absolute position, so in a situation of loss of reference, it causes the system to get lost.

There are different solutions that can be implemented to solve this problem. The most obvious would be to use another camera with a wider view of the work environment to determine the overall position; however, one might fear that it may introduce a lack of precision, and it would be necessary to implement another image processing algorithm. Furthermore, additional parts such as the second camera or its necessary support would make the micro-positioning system more complex and would not fulfill the original idea of using a system that is as efficient and cost-effective as possible.

Therefore, in order to maintain the same structure and hardware components of the original system to improve the global positioning system, a software modification is presented as the most suitable option.

Since a periodic and symmetrical pattern presents the aforementioned problem, it has been proposed to use an asymmetric and non-periodic pattern. Therefore, each image taken by the camera, regardless of the area of the image seen, is unique. Consequently, a logarithmic spiral pattern that includes the exposed conditions has been implemented. Spiral pattern analysis using the artificial vision algorithm will be more complex but will lead to better results.

**Figure 1:**
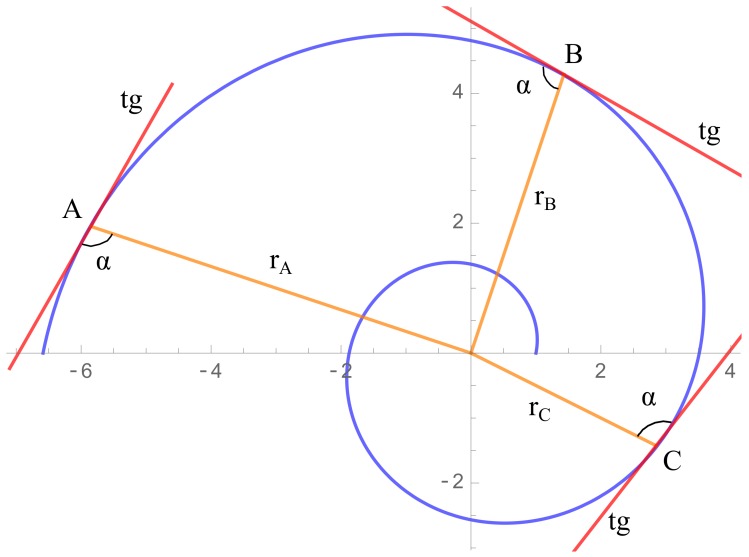
Representation of the constant angle α at any point of the logarithmic spiral.

The calculation method uses the properties of the logarithmic spiral such as the angle α built by the radius and tangent vectors at a point of the curve, remains constant all points of the curve as shown in Figure 1.

### 2.1. Method 1: Slope at the Point of Tangency

The camera used by the visual positioning system focuses on a specific area of the spiral shown on the screen, and this property is sufficient to calculate a global position. However, the center of the spiral must be approximated each time a movement is made. If the accuracy of the estimated central point is sufficient, the system may know its position. To do this, a simulation with a random spiral in Matlab will first be performed to validate the method; then, the spiral parameters will be discussed to find the values that best suit the real situation. As a starting point to simulate such a spiral, it is reminded that an arbitrary logarithmic spiral centered in a point $O=(x_{c},y_{c})$ is parametrized by Equation (1)

(1) $$\left\{\begin{array}{c} x\left(t\right)=ae^{bt}\cos\left(t\right)+x_{c}\\ y\left(t\right)=ae^{bt}\sin\left(t\right)+y_{c}, \end{array}\right.$$

where *x* is the abscissa of the spiral in a Cartesian coordinate system and *y* is the ordinate; *a* is the amplitude or scale factor; *b* is the divergence and *t* is the parameter to set the range of the spiral. In the presented case, the coordinates of the center $x_{c}$ and $y_{c}$ are randomly chosen by the algorithm.

**Figure 2:**
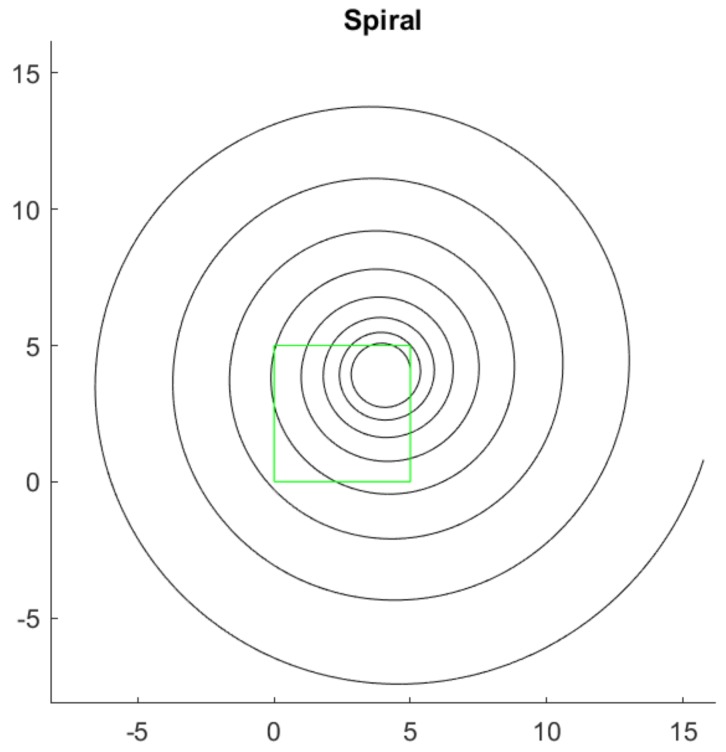
Method 1: Logarithmic spiral representation and analysis area (ROI). Axes in Matlab units.

Once the spiral has been generated, the first step of the method consist of taking the camera’s vision at an arbitrary portion of the spiral as shown in Figure 2, the green frame. This frame will be denoted as the *region of interest* (ROI). In the ROI, coordinates have been introduced in such a way that the origin (0,0) agrees with its lower left corner. This reference system is really important since the center of the spiral must be in the image reference system. In this way, all distances and coordinates will be expressed in the frame reference system (which simulates the image taken by the camera).

For the example shown in Figure 2, the values a=1, b=0.05, $x_{c}=4$, $y_{c}=4$ have been taken, obtaining a value of $\alpha =87.1376^{{}^{\circ}}$ by means of Equation (2)

(2) α=arctan(1/b).

**Figure 3:**
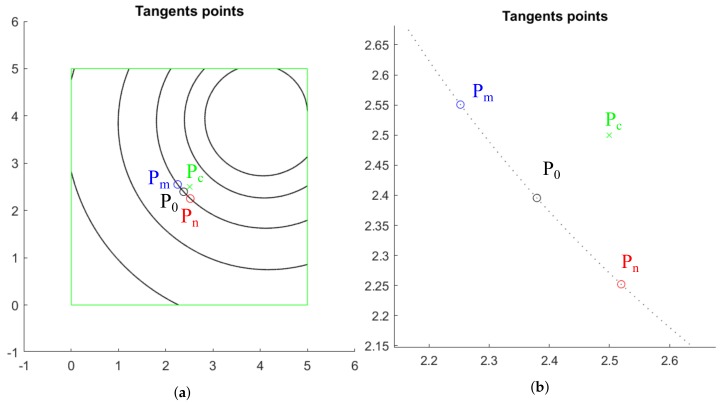
Logarithmic spiral: (**a**) ROI central point $P_{c}$ and the chosen $P_{0}$, $P_{m}$, $P_{n}$; (**b**) Enlargement to show the points gap between $P_{m}$ and $P_{n}$.

The second step consists in the reconstruction of the spiral only in the ROI, as shown in Figure 3, simulating the pixels as points in order to work without mathematical functions during this stage.

For the third step, two points are selected in the spiral, and two straight lines passing through these points that approximate the tangent lines at the spiral are drawn. To do this, a point $P_{0}$ is considered in the spiral having minimum distance *d* to the center of the ROI, denoted by $P_{c}$. Then, the points $P_{m}$ and $P_{n}$ are taken in the spiral at a distance of *d* points to $P_{0}$.

In the example shown in Figure 3a, the center $P_{c}$ has coordinates (2.5,2.5), and a distance of 20 points has been selected as shown in Figure 3b. Table 1 shows the *x* and *y* coordinates of the initial point $P_{0}$ on the curve and the points $P_{m}$ and $P_{n}$ where the approximation of the tangent lines will take place.

**Figure 4:**
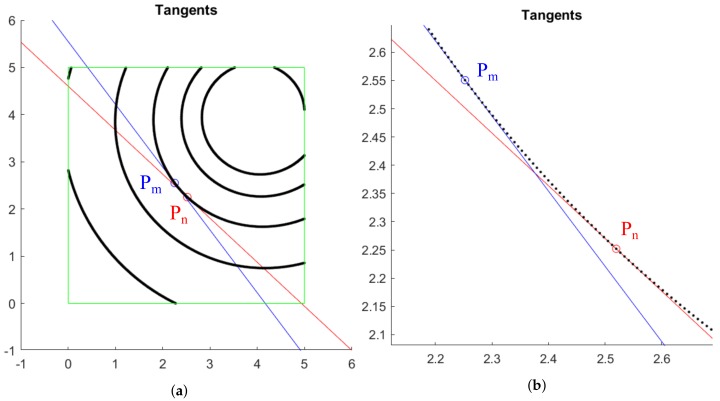
Approximated tangent lines at points $P_{m}$ and $P_{n}$: (**a**) General view throughout the ROI; (**b**) Enlargement over tangency points.

An approximation to the tangent line passing through $P_{m}$ is obtained by means of the well-known Equation (3)

(3) $$y_{tan}=\frac{dy*\left(x_{tan}-x\left(P_{m}\right)\right)}{dx}+y\left(P_{m}\right),$$

where $\left(x\left(P_{m}\right),y\left(P_{m}\right)\right)$ are the coordinates of the point $P_{m}$; dx and dy stand for the difference of the *x* and *y* coordinates between the points $P_{m}$ and its contiguous; and $y_{tan},x_{tan}$ are the ordinate and abscissa of the straight line. The same discussions hold for the point $P_{n}$. The straight lines passing through $P_{m}$ and $P_{n}$ are calculated as shown in Figure 4.

**Figure 5:**
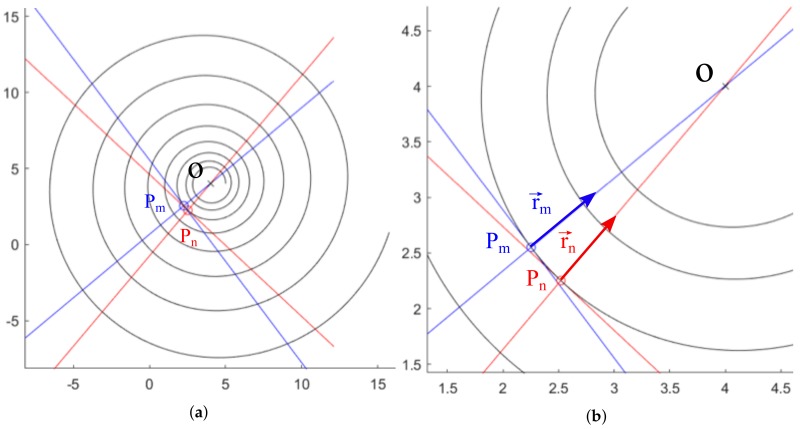
Lines with directions ${\vec{r}}_{m}$ and ${\vec{r}}_{n}$ that pass through $P_{m}$ and $P_{n}$, respectively: (**a**) General view; (**b**) Enlargement over the line drawing area.

Now, the line passing through $P_{m}$ (respectively through $P_{n}$) is rotated an angle α, obtaining the straight line with direction ${\vec{r}}_{m}$ (respectively the straight line with direction ${\vec{r}}_{n}$). The intersection between such lines, named $O_{1}$, will be the approximation to the center of the spiral *O*. See Figure 5 for a diagram of this construction.

### 2.2. Method 2: Compensated Slope

**Figure 6:**
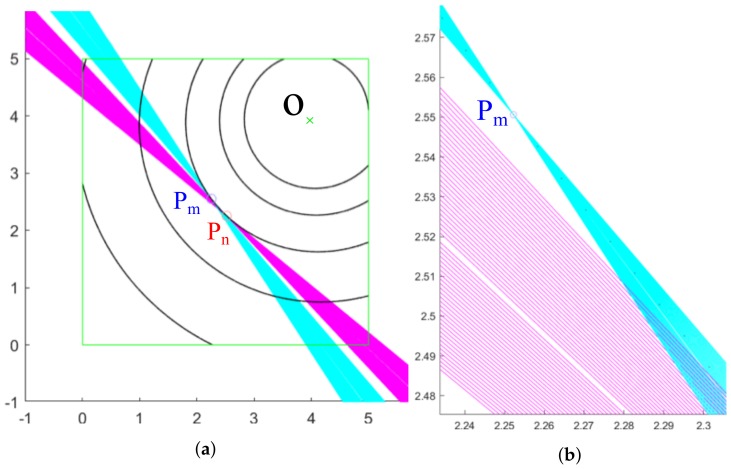
Lines adjacent to the points $P_{m}$ and $P_{n}$: (**a**) General view throughout the ROI; (**b**) Enlargement on the point $P_{m}$, showing the straight lines.

A second calculation method has been tested that relaxes the errors that could be made in the calculation of the tangent line with the information of a single point as performed by method 1. The goal remains the same, but the calculation method differs slightly. In this case, instead of calculating the tangents with Equation (3), lines that pass through one of the initial points ($P_{m}$ and $P_{n}$) are drawn but other adjacent points at both sides of $P_{m}$ and $P_{n}$ are used to generate more tangents lines to include its slopes as part of the final tangent calculation (Figure 6). One of the critical parameters of this method is, therefore, the determination of the number of lines to be drawn for the calculation (for the example shown, 100 lines have been selected for each of the points adjacent to $P_{m}$ and $P_{n}$). The aim of this method is to compensate for the error due to the discretization of the spiral in Matlab since the program uses independent points to generate the curve instead of a real continuous line.

At a later stage, a weighted average $\bar{m}$ of the slopes is calculated, providing more weight to the lines drawn with points closer to the starting point using Equation (4), where $m_{i}$ is the value of the slope calculated at point *i* and $w_{i}$ is the weight given to the slope at point *i* for the calculation of the average.

(4) $$\bar{m}=\frac{\sum_{i=1}^{n}m_{i}w_{i}}{n}.$$

Once the weighted slopes $\bar{m_{m}}$ and $\bar{m_{n}}$ at points $P_{m}$ and $P_{n}$ have been calculated, the tangent lines at these points are approximated using as tangent directions the slopes found. Next, it is proceeded as in method 1: the approximated tangents are rotated an angle α, and the intersection of such rotations is the approximated center $O_{2}$.

### 2.3. Comparison of Methods 1 and 2

**Figure 7:**
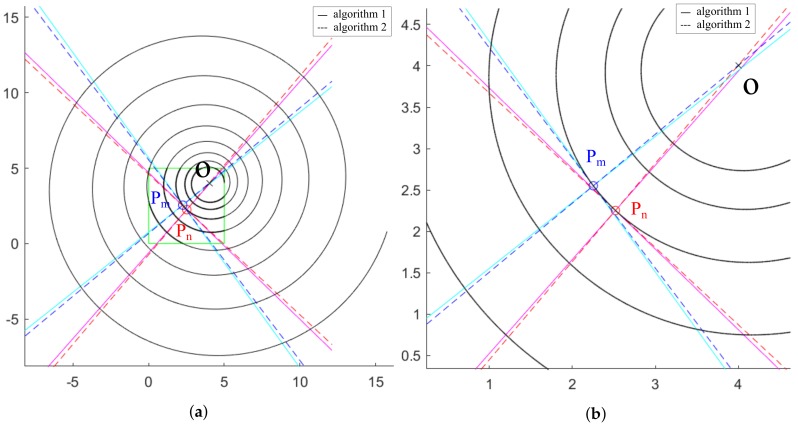
Methods 1 and 2 applied on points $P_{m}$ and $P_{n}$: (**a**) Overview; (**b**) ROI area.

Comparing the two exposed methods there are small differences in the approximated centers obtained, although both methods seem to be good approximations to the calculation (Figure 7).

**Figure 8:**
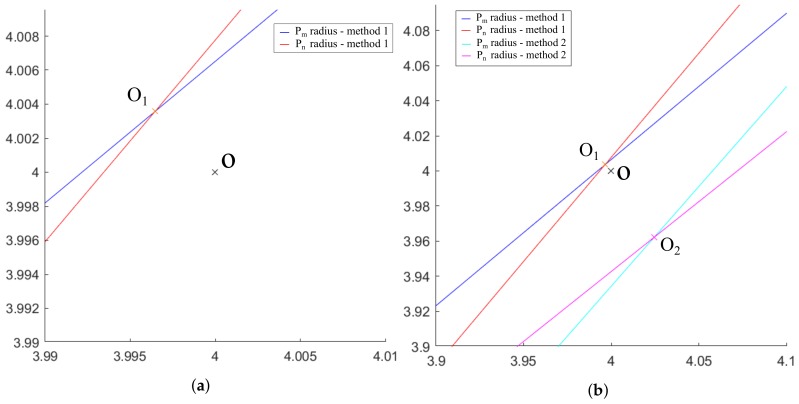
Approximations of the center of the spiral: (**a**) According to method 1; (**b**) Graphical comparison between method 1 and method 2.

With the selected parameters, the error in the calculation of the spiral center coordinates given by method 1 is represented in Figure 8a. This is compared graphically in Figure 8b in which the center is calculated as the intersection of the rotations of the tangents calculated at points $P_{m}$ and $P_{n}$ for the average of 60 slopes. Since the numerical value depends on the spiral creation parameters, it is not the most relevant data in this section. *O* designates the position of the real center of the spiral, $O_{1}$ indicates the position of the center of the spiral calculated according to method 1 and $O_{2}$ references the position of the center of the spiral calculated according to method 2.

**Figure 9:**
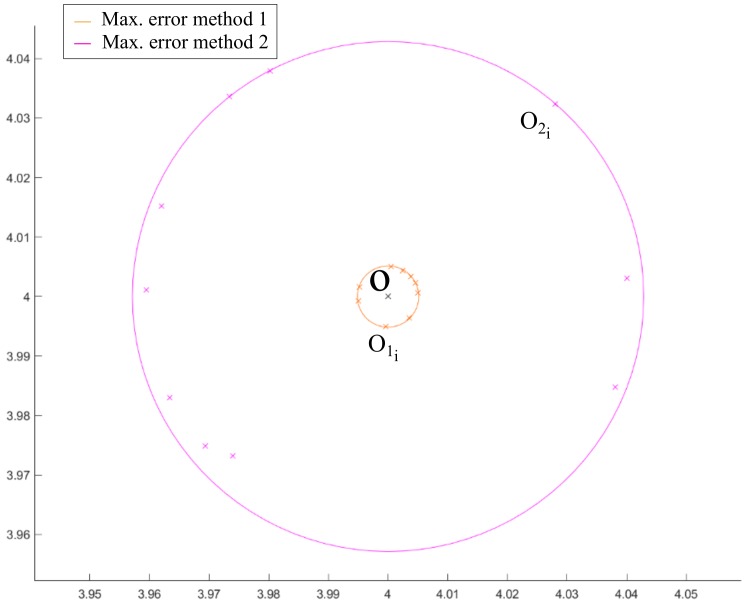
Comparison of the error made in the calculation of the spiral center using method 1 and 2 with 10 iterations.

The error provided by method 2 is greater than the one corresponding to method 1. Several simulations of the calculation of the spiral center have been carried out with both methods to verify stability, accuracy and precision. To do this, the calculations have been repeated 10 times and are represented in Figure 9, in which two circumferences have been drawn representing the maximum error found using each method, where $O_{1_{i}}$ with i=1,…,10, indicates the position of the centers of the spiral calculated according to method 1 for each iteration. Equivalently, $O_{2_{i}}$ with i=1,…,10, indicates the position of the centers of the spiral calculated according to method 2 for each of the iterations.

With the results obtained for the comparison of methods 1 and 2, the ratio between the radii of the maximum error circumferences shown in Figure 9 has a value of 8.33, confirming that method 1 makes an error of approximately 8 times less than method 2.

### 2.4. Parameters Influencing the Error

**Figure 10:**
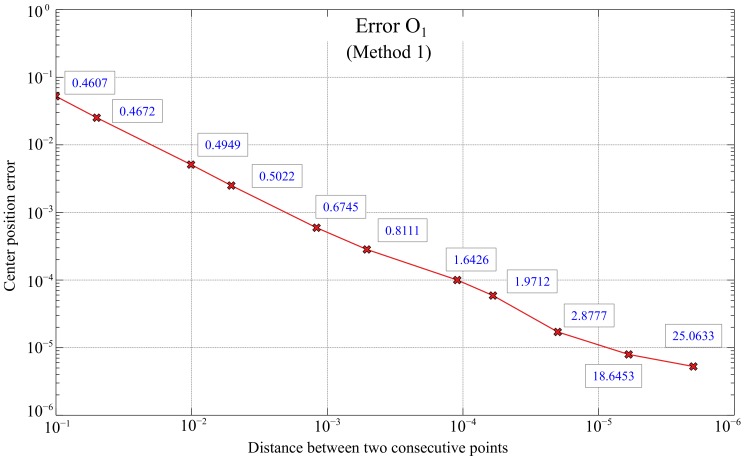
Errors in the calculation of the position of the center of the spiral ($O_{1}$) based on the distance between two consecutive points of the spiral (*d*) for method 1. The values next to each point represent the execution time of the algorithm (in seconds).

Method 1, which uses the rotation of the tangent line at a point in the spiral, the angle α known, provides better results than method 2, so it will be chosen as part of the final algorithm used for implementation in the global positioning system. The error depends on the accuracy when plotting the tangents, and therefore, on the distance *d* between the points used to calculate dx and dy in Equation (3). Given the importance of the *d* parameter, a simulation was carried out using 11 different values of distances between points to compare errors. Each point represented is the average of 100 iterations. The results are shown in Figure 10, whose axes are represented in logarithmic scale and Matlab units.

There is a clear relation between the error in the calculation of the center and the distance considered between two consecutive points when representing the spiral in Matlab. As expected, at a smaller distance *d* between points, the error when calculating the position of the center decreases, being a clear linear relation between the error and the distance between points.

In addition, the time, in seconds, required for the execution of the algorithm for each distance *d* is also represented in the same figure. That time does not increase linearly but much more slowly, so the selection of the appropriated *d* parameter will have to be done looking for the best compromise between the error and the calculation time.

### 2.5. Method 3: General System

The third method studied is based on the well-known Newton–Raphson method, which is a root search algorithm that uses tangents. Cheney [23] already analyzed the process of successively producing better approximations to the roots of a real valued function, but as Burden and Faires [24] already described in their numerical analysis of functions, the determination of the central point cannot be done exactly. Therefore, the error of the calculated coordinates of the center point must be evaluated. In the two previous methods the main drawback is that the spiral parameters must be known in advance. In fact, the value of the angle α must have been determined in advance because it is used by method 1 and method 2 to rotate the approximated tangent line vector and, consequently, be able to find the approximated center of the spiral. Therefore, it is necessary to develop a method allowing any type of logarithmic spiral to be used in the system, without prior knowledge of its parameters. In particular, the angle of rotation α is unknown.

As in the previous methods, the objective is to approximate the coordinates of the central point $O=(x_{c},y_{c})$ of the spiral.

**Figure 11:**
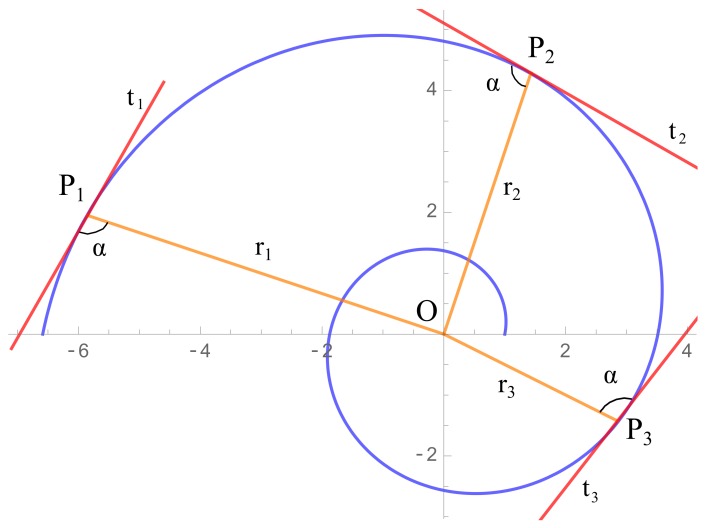
Elements and notation used in method 3.

Three points ($P_{1}$, $P_{2}$, $P_{3}$) are taken on the spiral of Figure 11, having coordinates $P_{i}=\left(x_{i},y_{i}\right),\;i=1,2,3$. We will denote by ${\vec{r}}_{i}=\vec{P_{i}O},\;i=1,2,3$ to the radius direction of the point $P_{i}$ to the center *O* of the spiral, by ${\vec{t}}_{i},\;i=1,2,3$ to the tangent vectors to the spiral passing through each $P_{i}$, and by α to the constant angle between ${\vec{r}}_{i}$ and ${\vec{t}}_{i}$. We emphasize here that no approximations have been made yet. Hence, the quantities $\alpha ,{\vec{t}}_{i},{\vec{r}}_{i}$ are related by using the definition of the scalar product as follows:

(5) $$\cos\alpha =\frac{<\vec{r_{1}},\vec{t_{1}}>}{\|\vec{r_{1}}\left\|\right\|\vec{t_{1}}\|}=\frac{<\vec{r_{2}},\vec{t_{2}}>}{\|\vec{r_{2}}\left\|\right\|\vec{t_{2}}\|}=\frac{<\vec{r_{3}},\vec{t_{3}}>}{\|\vec{r_{3}}\left\|\right\|\vec{t_{3}}\|}.$$

where

(6) $$\left\{\begin{array}{l} \vec{r_{1}}=\left(x_{c}-x_{1},y_{c}-y_{1}\right)\\ \vec{r_{2}}=\left(x_{c}-x_{2},y_{c}-y_{2}\right)\\ \vec{r_{3}}=\left(x_{c}-x_{3},y_{c}-y_{3}\right) \end{array}\right.$$

Substituting the values of Equation (6) into Equation (5), the following system of equations is obtained

(7) $$\begin{array}{ll} \cos\alpha & =\frac{<\left(x_{c}-x_{1},y_{c}-y_{1}\right)|\vec{t_{1}}>}{\|\left(x_{c}-x_{1},y_{c}-y_{1}\right)\left\|\right\|\vec{t_{1}}\|}\\ & =\frac{<\left(x_{c}-x_{2},y_{c}-y_{2}\right)|\vec{t_{2}}>}{\|\left(x_{c}-x_{2},y_{c}-y_{2}\right)\left\|\right\|\vec{t_{2}}\|}\\ & =\frac{<\left(x_{c}-x_{3},y_{c}-y_{3}\right)|\vec{t_{3}}>}{\|\left(x_{c}-x_{3},y_{c}-y_{3}\right)\left\|\right\|\vec{t_{3}}\|}. \end{array}$$

**Figure 12:**
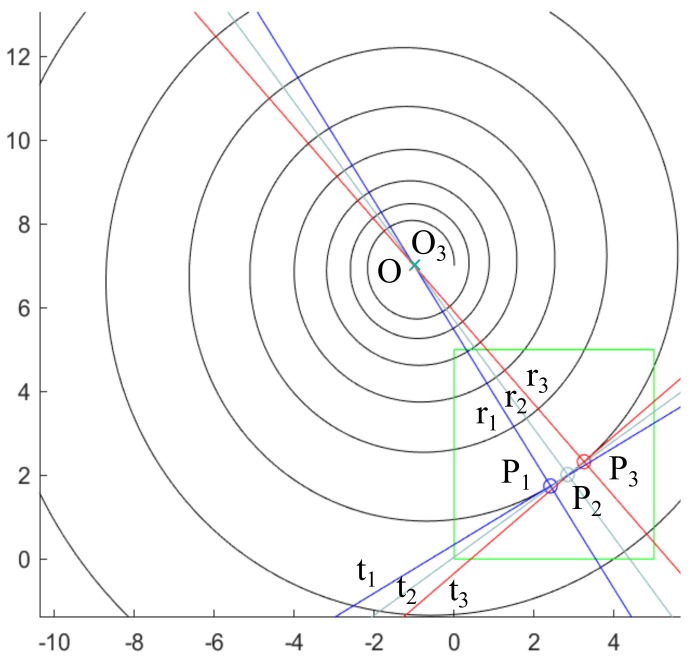
Graphical representation of method 3 with the approximated tangent and radial lines that pass through the points $P_{1}$, $P_{2}$ and $P_{3}$.

Note that system (7) has 9 indetermined values, namely, the center coordinates $x_{c},y_{c}$, the angle α and the coordinates of ${\vec{t}}_{i},\;i=1,2,3$. Thus, in order to give an approximate solution to this system of equations, the tangent values ${\vec{t}}_{i}$ will be approximated as done in method 1 (Section 2.1); these approximations will be named ${\vec{T}}_{i}$. Once the approximated tangents are introduced in Equation (7), a system of two equations and two variables ${\hat{x}}_{c}$ and ${\hat{y}}_{c}$ is obtained

(8) $$\begin{array}{l} \frac{<\left({\hat{x}}_{c}-x_{1},{\hat{y}}_{c}-y_{1}\right)|\vec{T_{1}}>}{\|\left({\hat{x}}_{c}-x_{1},{\hat{y}}_{c}-y_{1}\right)\left\|\right\|\vec{T_{1}}\|}=\frac{<\left({\hat{x}}_{c}-x_{2},{\hat{y}}_{c}-y_{2}\right)|\vec{T_{2}}>}{\|\left({\hat{x}}_{c}-x_{2},{\hat{y}}_{c}-y_{2}\right)\left\|\right\|\vec{T_{2}}\|},\\ \frac{<\left({\hat{x}}_{c}-x_{2},{\hat{y}}_{c}-y_{2}\right)|\vec{T_{2}}>}{\|\left({\hat{x}}_{c}-x_{2},{\hat{y}}_{c}-y_{2}\right)\left\|\right\|\vec{T_{2}}\|}=\frac{<\left({\hat{x}}_{c}-x_{3},{\hat{y}}_{c}-y_{3}\right)|\vec{T_{3}}>}{\|\left({\hat{x}}_{c}-x_{3},{\hat{y}}_{c}-y_{3}\right)\left\|\right\|\vec{T_{3}}\|}, \end{array}$$

where precisely, the variables ${\hat{x}}_{c}$ and ${\hat{y}}_{c}$ are the approximated coordinates of the expected center. That is, solving system (8) by means of Newton–Raphson’s method, an estimation $O_{3}=\left({\hat{x}}_{c},{\hat{y}}_{c}\right)$ to the center of the spiral *O* is obtained (Figure 12).

**Figure 13:**
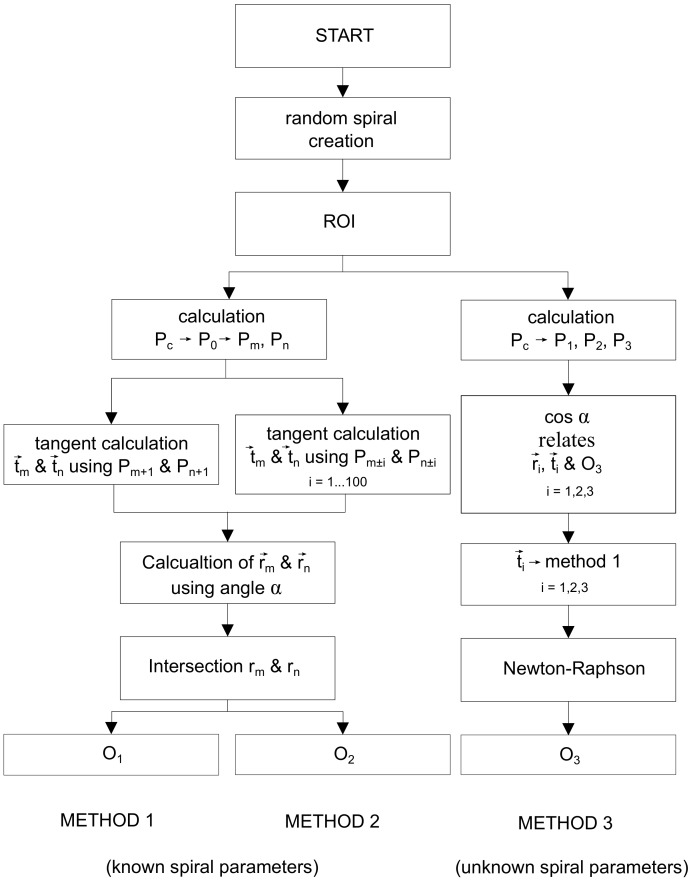
Flowchart methods 1-2-3.

**Figure 14:**
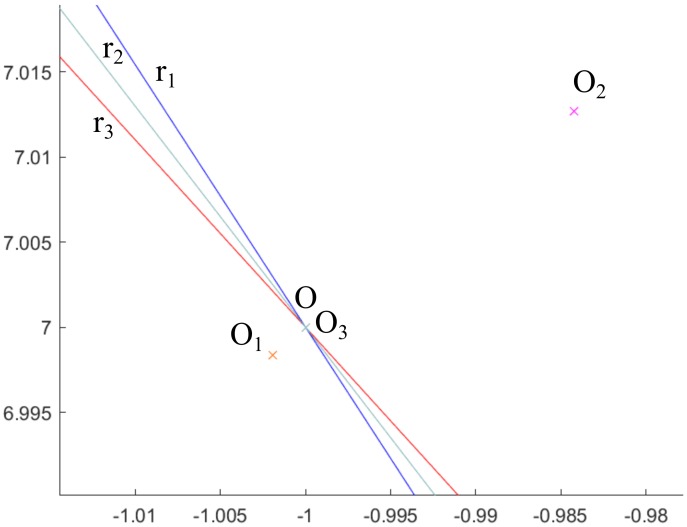
Comparison of the 3 methods showing $O_{1}$, $O_{2}$, $O_{3}$ and *O*.

The Figure 13 shows the Flowchart for the three methods presented. Additionally, the errors made in the determination of the spiral centers for the 3 proposed methods are shown in Figure 14 comparing graphically $O_{1}$, $O_{2}$, $O_{3}$ and *O*, clearly appreciating that method 3 is much more accurate than methods 1 and 2 (as it will be presented numerically below). Consequently, the error in method 3 is not distinguished in the mentioned figure, since it needs a much smaller graphic scale.

Since method 3 provides better results than the previous ones, it will be the method selected for implementation in the final global positioning system. Note that in this case the error depends only on the accuracy when estimating the tangents, and therefore, on the distance *d* between the points used to calculate dx and dy in Equation (3). For this reason, given the importance of the distance *d*, a simulation was carried out using 11 different values of distances between points in order to compare the errors made.

**Figure 15:**
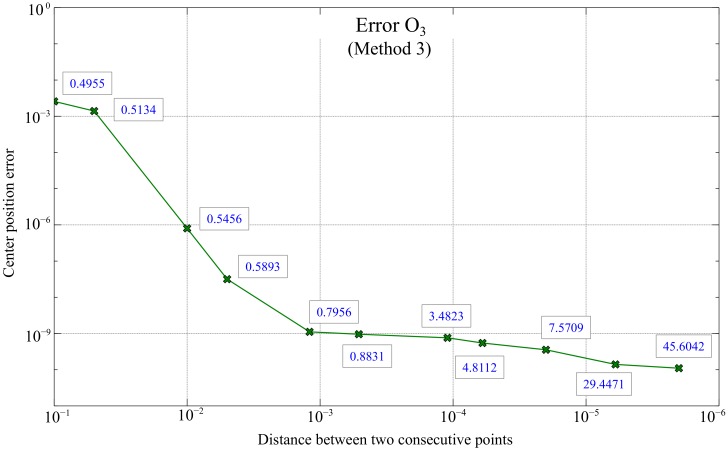
Errors in the estimation $O_{3}$ based on the distance *d* for method 3. The values next to each point represent the execution time of the algorithm (in seconds).

On the one hand, the results of the aforementioned simulation are shown in Figure 15, where each point represented is the average of 100 iterations and whose axes are represented in logarithmic scale and Matlab units. As expected, with these results it is confirmed numerically, the lower the distance *d* between points, the lower the error approximating the center. In fact, such relation seems to find an optimal point of distance *d* versus error when *d* is in the range of $10^{-3}$. Moreover, the time, in seconds, necessary for the execution of the algorithm for each distance *d* used is also included in Figure 15. It is observed that the time does not increase linearly but much more slowly, so the selection of the appropriate *d* parameter will be done looking for the best compromise between the error and the calculation time, similar to the previous methods presented.

On the other hand, the average errors, after 100 simulations with each distance *d*, made to estimate the center of the spiral *O* for each of the three methods are presented in Table 2. It should be noted that Matlab sometimes has difficulty solving system (8), particularly when *d* has a value below 0.001. Several times no solutions could be found even existing.

It is remarkable that these methods can provide large errors if the estimated tangent lines passing through different points of the spiral are almost parallel, since their intersection would be found very far away. As an example, during the simulations, some values found for $O_{1}$, $O_{2}$ and $O_{3}$ had an error of the order of $10^{3}$ with respect to the true center of the spiral when the points separated by a distance *d* were very close (below $10^{-1}$). However, this issue would be easily avoidable being careful when choosing the points in the spiral, that is, it should be checked that the selected points have direction vector of their tangent lines far from being parallel. Otherwise, newly revised points are selected taking into account this fact, and so the methods will work properly.

### 2.6. Actual Parameters, Experimental Results and Discussion

The results provided by the three methods presented have been carried out in Matlab with units and scales provided by the software itself, so the units are relative to the distances used. However, in order to implement any of these methodologies in a real positioning system, the true dimensions of the LCD screen where the logarithmic spiral will be displayed should be used. Therefore, the actual parameters must be taken into account. The LCD of the test device has a resolution of 1136×640 pixels and 326 ppi. In addition, the error expressed in generic Matlab units must be converted to millimeters to evaluate the accuracy of the method.

**Figure 16:**
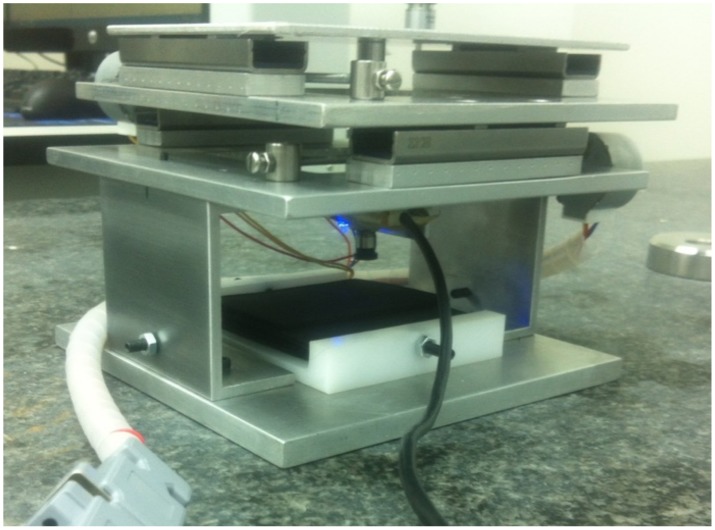
Micro-positioning test device for the calculation of the equivalences in mm of the study performed in Matlab.

The micro-positioning test device where the presented methods will be applied in a future phase of this project consists of a two-dimensional control system (Figure 16) [9] with two stepper motors (ST28, 12, 280 mA) which control and move two precision guides (IKO BSR2080 50 mm stroke) connected to a M3 ball screw/nut. The LCD screen used provides a 1136×640 pixel resolution, 326 ppi, and 0.078 mm dot pitch. The screen size was 88.5 × 49.9 mm. Both stepper motors are controlled by the digital output signals provided by an NI 6001-USB data acquisition card connected to the USB port of a laptop computer. The output signal of the acquisition card is treated by a pre-amplification power station composed of two L293 H-bridges. The control is programmed in LabVIEW. It receives the image captured by the camera and processes it according to an image enhancing process, which consists of an image mask application with color plane extraction, fuzzy pixel removal, small object removal, and particle analysis of the mass center of each evaluated pixel. Once the data of the image are processed using the artificial vision algorithm, they provide the positioning feedback signals needed to move the *x* and *y* axes on the test device platform. The images are taken by a digital camera (model MITSAI 1.3M, 1280×1024 pixels) included in the device.

Taking an amplitude or scale factor a=1 and a screen width of 640 pixels, it corresponds to 4.202453987724 units in the Matlab scale. Since 640 pixels have a width of 51.70 mm (width of the LCD screen used), each Matlab unit corresponds to 12.30233577 mm. The equivalence between both systems (Matlab units vs. Display units) is shown in Table 3.

The consistency in the determination of the center of the spiral is tested with 1000 iterations using method 3. The average error *e* and the standard deviation *s* of the sample are presented in Table 4. The parameters of the spiral are: a=1, b=0.05, d=0.05 mm.

Therefore, it has been possible to develop an absolute positioning system with an error below 1 mm without using very small values of the distance between points *d*, being the algorithm accurate and fast. However, the disparity in the results is remarkable. The non-precision or high distribution of them around the average value is clearly reflected in the value obtained for the standard deviation with a value close to 53% of the average value itself. This effect has been minimized by increasing the number of iterations in each simulation. Consequently, although the average value provides a valid number, in the future, it will be necessary to improve the algorithm to achieve better stability and accuracy.

**Figure 17:**
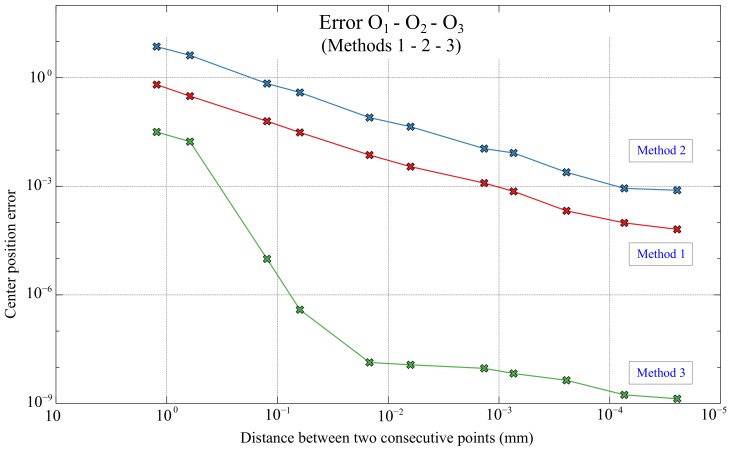
Errors in the estimations $O_{1},O_{2},O_{3}$ based on the distance *d* for methods 1, 2 and 3 in mm.

The errors, in mm, made by each of the three methods shown in Table 5 and in Figure 17 highlight, once again, the great difference in accuracy of method 3 with respect to methods 1 and 2. In addition, once the Matlab scale has been converted to the scale used in the positioning system, it is observed that the errors made in the calculation of the spiral center position with method 3 are below 1 μm just selecting the distance values *d* below 0.12 mm.

## 3. Discussion and Conclusions

A global reference system is a requirement in any positioning system that aims to provide robustness and stability. Therefore, absolute reference is a key factor in precision positioning systems since a loss of reference causes a reduction in accuracy and precision. Because of this, in this work, three different methods have been presented to achieve an absolute positioning system based on a vision system in which an asymmetric pattern in the form of a logarithmic spiral has been used. This pattern is different from the one used for the development of the camera-screen system presented in previous works [8] based on a pattern of illuminated repetitive LEDs on the screen in which the position calculation was always based on the relative position with respect to the previous point calculated. Such a method, in case of mechanical errors in the system, could provide multiple position solutions, causing the loss of reference.

The three methods presented are based on the property of the logarithmic spiral in which the tangent line at any point of the curve and the line that passes through the center of the curve from that point form a constant angle. The algorithms have been tested by performing different simulations in Matlab, taking random images of an area of the logarithmic spiral represented and calculating the position of the center of the spiral. If the center position is known, the system is able to position itself with respect to the rest of the system, achieving the desired absolute positioning system. All the studied algorithms present good solutions for the calculation of the spiral center position. However, methods 1 and 2 need to know the constant angle of the spiral for the calculation of the center; whereas, method 3, based on the Newton–Raphson method, in addition to not requiring any of the spiral parameters to be known in advance, is the method that provides the smallest errors in the calculation of the center position. Since the errors depend on the discretization of the curve in its simulation that is defined by the distance between points of the spiral used, different simulations have been made finding that, with a distance of 0.05 mm between points on the curve, method 3 is able to position the center of the spiral with an error of less than $1\times 10^{-6}$ mm. The novelty of this work is, on the one hand, a new approach to a global positioning system using the non-periodic pattern of a logarithmic spiral and, on the other hand, the reduction of the error in the determination of the global positioning which can be implemented in a micro positioning system. 

## Figures and Tables

**Table 1:** Coordinates *x* and *y* of the selected points to plot the approximated tangents in method 1.

Coordinate	$P_{m}$	$P_{0}$	$P_{n}$
*x*	2.25185	2.38209	2.52098
*y*	2.54879	2.39567	2.25308

**Table 2:** Errors made by each of the 3 methods for the estimation of *O* based on the distance *d*.

*d*	Error $O_{1}$	Error $O_{2}$	Error $O_{3}$
0.1002	0.0523	0.5876	2.578 × 10${}^{-3}$
0.0501	0.0252	0.3372	1.393 × 10${}^{-3}$
0.0101	0.0051	0.0561	8.013 × 10${}^{-7}$
0.0051	0.0025	0.0318	3.168 × 10${}^{-8}$
0.0012	5.942 × 10${}^{-4}$	6.431 × 10${}^{-3}$	1.106 × 10${}^{-9}$
5.113 × 10${}^{-4}$	2.835 × 10${}^{-4}$	3.602 × 10${}^{-3}$	9.548 × 10${}^{-10}$
1.102 × 10${}^{-4}$	1.000 × 10${}^{-4}$	8.903 × 10${}^{-4}$	7.602 × 10${}^{-10}$
6.008 × 10${}^{-5}$	5.897 × 10${}^{-5}$	6.788 × 10${}^{-4}$	5.460 × 10${}^{-10}$
2.003 × 10${}^{-5}$	1.707 × 10${}^{-5}$	1.985 × 10${}^{-4}$	3.551 × 10${}^{-10}$
6.008 × 10${}^{-6}$	7.934 × 10${}^{-6}$	7.140 × 10${}^{-5}$	1.403 × 10${}^{-10}$
2.003 × 10${}^{-6}$	5.264 × 10${}^{-6}$	6.317 × 10${}^{-5}$	1.101 × 10${}^{-10}$

**Table 3:** Equivalence between Matlab units and display units in mm.

Matlab Units	Equivalence in mm
4.202453987724	51.7
0.0812853769385687	1
1	12.30233577

**Table 4:** Average error *e* and standard deviation *s*, in millimeters, between the real center and the center provided by method 3 using as spiral parameters a=1, b=0.05, d=0.05 mm in a simulation of 1000 iterations.

Statistical	Error
Parameter	$\times 10^{-6}$ mm
***e***	**0.3996**
*s*	0.2188

**Table 5:** Errors, in mm, made by each of the 3 methods for the estimation of *O* based on the distance *d*.

*d*	*d*	Error $O_{1}$	Error $O_{2}$	Error $O_{3}$
(Matlab)	(mm)	(mm)	(mm)	(mm)
0.100200000	1.232694044	0.643412161	7.228852498	0.0317104638
0.050100000	0.616347022	0.310018861	4.148347621	0.0171334753
0.010100000	0.124253591	0.062741912	0.690161035	0.0000098582
0.005100000	0.062741912	0.030755839	0.391214275	0.0000003897
0.001200000	0.014762803	0.007310417	0.079113369	0.0000000136
0.000511270	0.006289815	0.003487343	0.044308583	0.0000000117
0.000110170	0.001355348	0.001230725	0.010953013	0.0000000094
0.000060084	0.000739174	0.000725494	0.008350535	0.0000000067
0.000020026	0.000246367	0.000210014	0.002442264	0.0000000044
0.000006008	0.000073908	0.000097597	0.000878375	0.0000000017
0.000002003	0.000024635	0.000064745	0.000776931	0.0000000014

## Abbreviations

The following abbreviations are used in this manuscript:

LCD	Liquid Cristal Display
CNC	Computer Numerical Control
ROI	Region of Interest
